# Trial data management: lessons from a large orthopedic trial

**DOI:** 10.1186/1745-6215-14-S1-P141

**Published:** 2013-11-29

**Authors:** Erik Lenguerrand, Vikki Wylde, Rachael Gooberman-Hill, Sian Noble, Elsa Marques, Paul Dieppe, Ashley Blom

**Affiliations:** 1 University of Bristol, Bristol, UK; 2 University of Exeter, Exeter, UK

## Background

We conducted two double-blinded RCTs of peri-operative pain management for patients undergoing hip(N=318) or knee replacement(N=319). Intervention groups received an additional injection of local anaesthetic and steroid into the soft tissues around the joint during surgery and control groups received treatment as usual. Data were collected pre-operatively, during surgery, during the inpatient period, and 3, 6 and 12months post-operatively.

Data quality in RCTs is essential to ensure robust results. This is particularly challenging in multidisciplinary trials with multiple follow-ups and diverse sources of information. This paper describes practices designed to maximize data quality and reflects on optimal practice in trial conduct.

## Methods

Data were collected using clinical examinations, self-report questionnaires and extraction from medical notes. The project was supported by methodologists(statistician/data-manager, health economists, patient-involvement and qualitative researchers), clinicians(surgeons and anesthetists), research nurses, a trial manager and an administrator. Data was inputted into Access databases, with data quality checks conducted in STATA(12.1).

## Findings

Monthly double data-entry conducted on random subsets of patients ensured ongoing accuracy(~99.5%). Losses to follow-up(<2%) and missing data were minimized by active monitoring of data collection and completeness. Structured query language and descriptive statistics were used to investigate data quality within and across collection points. Regular meetings were held between staff from various disciplines to facilitate interpretation of any inconsistencies in clinical data or departure from the SOPs.

## Conclusion

To ensure rigor within orthopedic RCTs, staffing and sufficient allocation of resources to data quality are paramount. Data-cleaning processes should be considered proactively in the early stages of trial design and conduct.

